# Melatonin Represses Mitophagy to Protect Mouse Granulosa Cells from Oxidative Damage

**DOI:** 10.3390/biom11070968

**Published:** 2021-06-30

**Authors:** Yi Jiang, Ming Shen, Yuanyuan Chen, Yinghui Wei, Jingli Tao, Honglin Liu

**Affiliations:** College of Animal Science and Technology, Nanjing Agricultural University, Nanjing 210095, China; 2017205014@njau.edu.cn (Y.J.); shenm2015@njau.edu.cn (M.S.); 2019105022@njau.edu.cn (Y.C.); 2019105047@njau.edu.cn (Y.W.)

**Keywords:** melatonin, oxidative damage, mitophagy, granulosa cells, PINK1-Parkin pathway

## Abstract

Various environmental stimuli, including oxidative stress, could lead to granulosa cell (GC) death through mitophagy. Recently, it was reported that melatonin (MEL) has a significant effect on GC survival during oxidative damage. Here, we found that MEL inhibited oxidative stress-induced mitophagy to promote GC survival. The loss of cell viability upon H_2_O_2_ exposure was significantly restored after MEL treatment. Concomitantly, MEL inhibited the activation of mitophagy during oxidative stress. Notably, blocking mitophagy repressed GC death caused by oxidative stress. However, MEL cannot further restore viability of cells treated with mitophagy inhibitor. Moreover, PTEN-induced putative kinase 1 (PINK1), a mitochondrial serine/threonine-protein kinase, was inhibited by MEL during oxidative stress. As a result, the E3 ligase Parkin failed to translocate to mitochondria, leading to impaired mitochondria clearance. Using RNAi to knock down PINK1 expression, we further verified the role of the MEL-PINK1-Parkin (MPP) pathway in maintaining GC survival by suppressing mitophagy. Our findings not only clarify the protective mechanisms of MEL against oxidative damage in GCs, but also extend the understanding about how circadian rhythms might influence follicles development in the ovary. These findings reveal a new mechanism of melatonin in defense against oxidative damage to GCs by repressing mitophagy, which may be a potential therapeutic target for anovulatory disorders.

## 1. Introduction

In mammalian ovaries, follicular atresia is a physiological phenomenon that destroys more than 99% of the follicles [1,2]. Atresia-derived anovulation is closely related to some reproductive disorders, such as polycystic ovary syndrome and primary ovarian insufficiency [3]. It has been reported that follicular atresia is attributed to the programmed cell death of ovarian granulosa cells (GCs) [4]. Reactive oxygen species (ROS) is a class of highly reactive radicals that are normally produced in the course of oxygen metabolism [5]. However, excessive ROS generation causes oxidative damage to cellular components [6]. Reproduction is an energy demand process associated with accelerated metabolism rates and ROS production [6]. Previous studies showed the participation of oxidative stress in triggering follicular atresia by inducing GC apoptosis or autophagy [7], although the underlying mechanism remains to be investigated. The growth and development of follicles includes follicle formation, follicle development and follicle atresia. Ovogenesis is inseparable from the growth and maturation of follicles. Primary oocytes are formed from the proliferation and differentiation of the primordial germ cells located in the genital ridges during early embryonic development. After the formation of primary oocytes, they combine with the surrounding monolayer follicular cells and gradually evolve into primitive follicles. Under the action of unknown mechanism, the primitive follicle ends dormancy state and enters the basal growth phase. The growth and development of follicles mainly include primary follicles, primary follicles, secondary follicles, tertiary follicles and Graff’s follicles. However, only a very small number of follicles complete the development process. Most follicles may degenerate and disappear during each period of growth and development, which is called follicular atresia. Gonadotropins, ovarian sex steroid hormones and other ovarian local factors play a key role in the regulation of follicle survival. Previous studies have shown that follicular atresia is caused by granulosa cell apoptosis. A follicle is a tiny vesicular structure in the ovarian cortex of a female animal, consisting of an oocyte and surrounding somatic cells. The shape, size and distribution of follicles in the ovary depend on the species of the animal and the physiological stage of the animal. The growth of follicles is accompanied by a series of changes in follicle morphology and structure. The total number of follicles is fixed before the animal is born. Therefore, during ontogeny, follicular storage decreases with follicular atresia and ovulation. Follicle is an important place for the ovary to store oocytes, so the quality of follicle development directly affects the subsequent ovulation and fertilization. For female mammals, oxidative stress has an adverse effect on the normal development of the ovary and is an important cause of follicular atresia. In mammals, most follicles atresia during development, and in humans more than 99.9% of the follicles die by atresia. Even in rodents with high utilization, no more than 1% of the eggs end up as offspring. At present, follicular atresia is mainly a process of granulosa cells undergoing large-scale apoptosis, and from the perspective of morphological changes, the occurrence of granulosa cells apoptosis is far earlier than the occurrence of follicular atresia, and the phenomenon of follicular atresia can only be observed when the apoptotic granulosa cells in the follicle reach a certain level. Therefore, granulosa cell apoptosis is considered to be the initiator of follicular atresia. Although most follicles go to atresia and die during development, some develop to maturity and ovulate.

Melatonin (N-acetyl-5-methoxytryptamine, MEL), an amine hormone, is synthesized by the pineal gland and many other organs [8]. Since the discovery of melatonin, a large number of studies have confirmed that it has the physiological functions of improving sleep, delaying aging and scavenging ROS [9]. In the ovary, MEL serves as a pro-survival factor of GCs and antral follicles [10]. It facilitates GCs to generate estrogen, elevates the growth of antral follicles and regulates the selection and dominance of preovulatory follicles [11,12,13]. As an effective antioxidant, MEL was supposed to antagonize oxidative stress-induced GC damage [7,14]. However, the downstream signaling of MEL for GC protection remains largely undetermined. In the process of follicle development, melatonin concentration increases with the enlargement of follicle volume, suggesting that melatonin plays an important role in the process of follicle development. On the one hand, melatonin can stimulate the secretion of gonadal hormone, stimulate the proliferation of follicular granulosa cells and promote the development of follicles. On the other hand, melatonin clears the follicles of molecules that cause oxidative stress, such as ROS. In addition, melatonin has been found in mammalian follicular fluid. Therefore, melatonin plays an important role in follicular development. In order to study the effect of melatonin on hypoxia-induced granulosa cells of follicles, this study added exogenous melatonin under hypoxia-induced conditions, and explored the alleviation of melatonin on hypoxia-induced granulosa cells apoptosis and its mechanism.

During follicular atresia, several forms of programmed cell death (PCD), including autophagy, were detected in ovarian GCs [15,16]. Recent evidence revealed that autophagy might be responsible for oxidative stress-induced GC damage [17,18]. For example, GC death was reported to be induced by oxLDL-dependent activation of autophagy [17,18]. Interestingly, obese women who had high levels of oxLDL also showed higher level of oxidative stress in the ovary [18], resulting in increased ration of anovulatory infertility. This prompt us to ask whether MEL-mediated GC protection is correlated with autophagy regulation.

Autophagy could be classified into several types depending on the specific degradation of cellular organelles. Particularly, mitophagy is the selective degradation of mitochondria by autophagy [19]. Accumulating evidence implied that increased permeability of mitochondrial membrane during oxidative stress stimulates mitochondrial membrane potential (Δψm) depolarization, leading to PTEN-induced kinase 1 (PINK1) accumulation on the outer mitochondrial membrane [20]. Then, the E3 ubiquitin ligase Parkin was recruited to damaged mitochondria, which triggers the mitophagic degradation [21]. However, mitophagy signaling pathways display distinct roles in different cell types or stress conditions [22]. The probable involvement and molecular mechanisms of mitophagy in the MEL-mediated preservation of GCs against oxidative stress have not been investigated methodically. In this study, we investigated the protective effects of MEL on GCs from different aspects, including cell viability, molecular component of mitophagy signaling, mitochondrial integrity and mitophagy flux. The results showed that MEL protects GCs from oxidative damage by repressing PINK1-Parkin-dependent mitophagy.

## 2. Materials and Methods

### 2.1. Cell Culture

The primary GCs were isolated as described [23]. First, intraperitoneally injected into female ICR mice aged 3–4 weeks with pregnant horse serum gonadotropin (PMSG). After 48 h, the cervical vertebra was dislocated and killed. Under aseptic conditions, bilateral ovaries were quickly removed and immediately placed in PBS in a small petri dish (35 × 15 mm^2^). The process is as follows: puncture large follicles (diameter > 200 μm, antral follicles) with a 1 mL syringe needle under anatomic microscope and release granulosa cells, pull out granulocyte cells a certain distance from the ovary, try to prevent/stop cell contamination, accumulate a certain amount of granulosa cells, use a pipette to suck them out, then transfer them to 15% serum (purchased from Gibco, New York, NY, USA), 100 U/mL penicillin (purchased from Gibco), 100 μg/mL streptomycin (purchased from Gibco) and DMEM/F-12 (1:1) (purchased from Invitrogen, Carlsbad, CA, USA).

For different purposes, inoculate the cells on a 6-well plate, a T25 culture flask, a T75 culture flask or a cell slide cultures in a 37 °C cell incubator containing 5% CO_2_, changing the medium every 2–3 days. After 4–6 days, the fusion rate of granulosa cells is about 90% (the number of cells is 5 × 10^5^ in the one hole of a 6-well plate), which can be used in various drug treatment experiments. For drug administration, GCs pre-treated with 200 μM H_2_O_2_ (sigma, St. Louis, MO, USA) for 1 h are washed in PBS and incubated with 10 μmol/L melatonin for 2 h.

### 2.2. Autophagy Detection

The formation of acidic vesicular organelles (AVOs), an indicator of autophagy, was detected using acridine orange staining. After the desired treatments, GCs were stained with 1 μg/mL acridine orange for 30 min at 37 °C. With the flow cytometric assay, the emission of green (510–530 nm) and red (650 nm) fluorescence filter (BD Accuri C6, Piscataway, NJ, USA) was used. 

### 2.3. Cell Viability Assay

The Cell Counting Kit-8 (CCK8, APExBIO, Houston, TX, USA) was used to analyze cell viability. Cells were seeded in 96-well plates and exposed to the treatments as indicated. Then, 10 μL of CCK-8 solution were added to each well for 4 h at 37 °C in the dark. Subsequently, the samples were analyzed by reading the optical density at 450 nm using a microplate reader (Bio-Rad; Hercules, CA, USA).

### 2.4. Western Blot Analysis

GCs were lysed with ice-cold RIPA Lysis Buffer (Beyotime, P0013B, Shanghai, China) plus a complete protease inhibitor cocktail (Roche, 04693132001, Basel, Switzerland), and protein concentrations were determined using a BCA Protein Assay Kit (Beyotime, P0012, Shanghai, China) according to the manufacturer’s instructions. Whole cell lysates were prepared by 5×SDS loading buffer, and then boiling for 10 min. Western blotting assays were performed as described previously [22]. Briefly, the pretreated protein samples were separated by SDS-PAGE and transferred to PVDF membranes (Millipore, Billerica, MA, USA) by electroblotting. The membranes were incubated with 4% (*w*/*v*) non-fat mike powder for 2 h at room temperature to block the non-specific binding sites. Anti-LC3B, anti-P-mTOR, anti-P62, anti-caspase-3, anti-TOM and anti-PINK1 antibodies were used as primary antibodies and incubated overnight. Then, the membranes were washed by TBST and incubated with secondary antibody for 2 h at room temperature. Bands were visualized by using super ECL plus (Thermo Fischer Scientific, Waltham, MA, USA) and analyzed by Image J software (version 1.45; National Institutes of Health, Bethesda, MD, USA). Each experiment was repeated at least 3 times, and representative data are shown.

### 2.5. Tetramethyl Rhodamine Ethyl Ester (TMRM) Staining Assays

To evaluated cell health and mitochondrial function, tetramethyl rhodamine ethyl ester (TMRM) staining assays was performed. Cells were seeded in 12-well plates and grown with different treatments for 24 h. After removing the culture medium, cells were incubated with TMRM staining solution for 30 min at 37 °C. For FCM (flow cytometry) analysis, a 488 nm laser was used to detect TMRM emission at 570 ± 10 nm. 

### 2.6. Mitochondrial Membrane Potential Assay (JC-1 Staining)

Mitochondrial membrane potential was monitored by a mitochondrial-specific dual fluorescence probe, JC-1. Briefly, JC-1 was added to reach a final concentration of 5 mg/mL. After incubation for 20 min, the cells were washed twice with medium, and detected by flow cytometry at 488 nm excitation wavelength to detect JC-1 emitted light from 530 nm to 590 nm.

### 2.7. Electron Microscopy

PBS-washed cells were collected into microcentrifuge tubes using cell scrapers. After centrifugation, 2.5% (*v*/*v*) glutaraldehyde was added carefully on cell pellets for an overnight fixation at 4 °C. Fixed cells were further post-fixed with 1% osmium tetroxide, dehydrated and embedded in Araldite. After being sliced, the sample was mounted on Formvar-coated grids and then the images of their ultrastructure were visualized under transmission electron microscopy (TEM; Hitachi, Tokyo, Japan, H-7650; Hitachi, Tokyo, Japan).

### 2.8. Mitophagy Detection by MT-Keima Transfection

Mitochondria-targeted keima (MT-Keima) was employed to monitor mitophagy since keima can emit various-colored signals in neutral and acidic environments [24]. In the present study, GCs were seeded on coverslips in 12-well plates, grown to 60–80% confluence, and transfected with pMT-mKeima-Red (AM-V0251, MBL, Nagano-ken, Japan) using Lipofectamine 3000 (Invitrogen). Twenty-four hours later, cells were treated with H_2_O_2_ or MEL. The cells were then washed twice with PBS and detected the fluorescence signal excited by 586 nm excitation light by FCM. All experiments were repeated three times.

### 2.9. RNA Interferencen

siRNA specific for PINK1 (sc-44599) and its scrambled controls (sc-37007) or Parkin (sc-32282) and its scrambled controls (sc-32282) were all purchased from Santa Cruz Biotechnology (Santa Cruz, CA, USA). siRNA transfections were performed using Lipofectamine 3000 reagent (Invitrogen) according to the manufacturer’s instructions.

### 2.10. Immunofluorescence

After treatment, GCs were fixed with 4% paraformaldehyde for 1 h, permeabilized using 0.5% Triton X-100 in PBS for 10 min at 4 °C, and blocked with BSA for 2 h at room temperature. The GCs were incubated with rabbit anti-PINK1 (1:100; Santa Cruz, CA, USA) primary antibodies, rabbit anti-Parkin (1:100; Santa Cruz) or mouse anti-Tom20 (1:100; Santa Cruz) overnight at 4 °C, and then incubated with rabbit or mouse fluorescent secondary antibodies (1:200; Invitrogen) for another 1 h at 37 °C. The coverslips were washed, mounted in citifluor containing 4′,6-diamidino-2-phenylindole and observed with confocal microscope (Zeiss LSM 710 META, Oberkochen, Germany).

### 2.11. Statistical Analysis

All data in our experiment were presented as mean ±S.E. The statistical analysis was performed with SPSS version 16.0 software. One-way ANOVA was used to analyze the significance of the differences between the groups. All experiments were repeated at least three times. A value of *p* < 0.05 was considered statistically significant.

## 3. Results

### 3.1. Melatonin (MEL) Inhibits the Initiation of Granulosa Cells (GCs) Autophagy under Oxidative Stress

**Figure 1:**
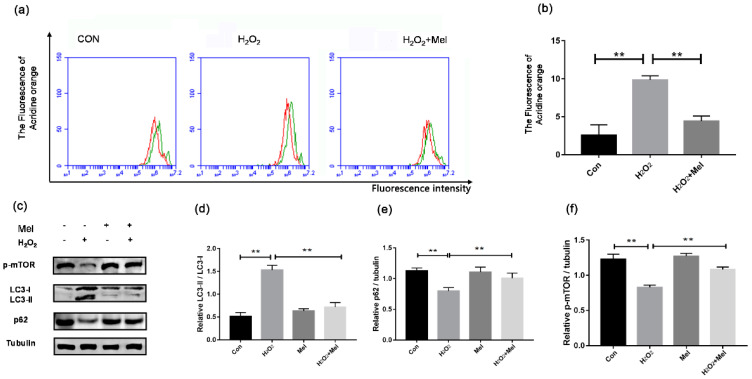
MEL suppressed the oxidative stress-induced autophagy of GCs. Primary cultured GCs were pre-treated with H_2_O_2_ (200 μM) for 1 h, and then treated with MEL (10 μmol/L) for another 2 h. The acidic autophagic vacuoles were detected using acridine orange staining and detected by FCM, and the LC3 protein was detected by Western blotting with anti-LC3. The +Y axis is 100 times the number of cells, and the + X axis is the intensity of cell fluorescence. (**a**,**b**) The fluorescence degree of acridine orange. (**c**–**f**) Immunoblot assay for the expression of LC3, p62 and p-mTOR in GCs. α-tubulin served as the control for loading. Experiments were repeated in triplicate. Data represent mean ± S.E; *n* = 3. ** Represents *p* < 0.01 compared to control group.

To test whether MEL influences GC autophagy during oxidative stress, acridine orange staining was adopted as an indicator in flow cytometry to detect the wavelength of acidic autolysosomes. As shown in Figure 1, H_2_O_2_ treatment obviously increased the autolysosomes number in cultured GCs, while MEL markedly reduced the autophagic signal in H_2_O_2_-treated cells (Figure 1a). Consistent with this, the activation of some autophagy biomarkers upon H_2_O_2_ exposure was suppressed in the presence of MEL. The results of Western blotting analysis showed that LC3-II accumulation, p62 degradation, mTOR dephosphorylation and the conversion of LC3-I to LC3-II were inhibited when H_2_O_2_-treated GCs were cultured with MEL (Figure 1b–e). 

### 3.2. MEL Protects GCs from Oxidative Damage by Repressing Autophagy 

**Figure 2:**
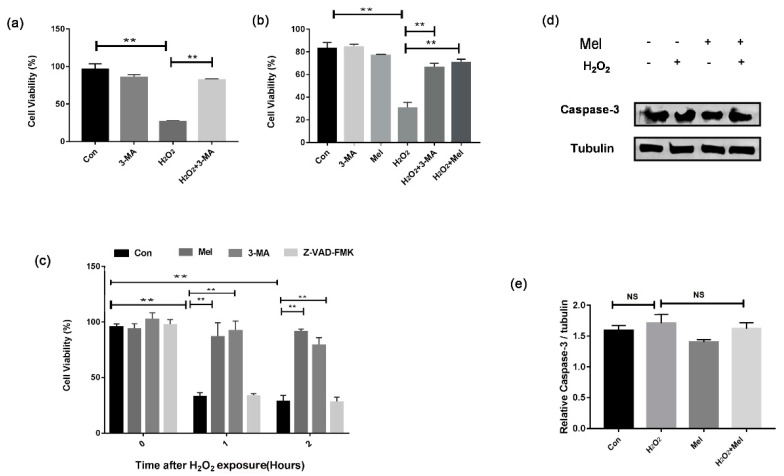
MEL protects GCs from oxidative damage by suppressing autophagy. (**a**–**c**) Primary GCs were treated with H_2_O_2_ (200 μ M) for 1 h, and then cultured with 10 μM MEL, 50 μM 3-MA or 50 μM Z-VAD-FMK, and 0, 1 or 2 h later, cell viability was detected using the CCK-8 assay. (**d,e**) GCs were incubated with 200 μM H_2_O_2_ for 1 h, then treated with or without MEL for 2 h. The expression of caspase-3 in GCs was determined by Western blotting. α-tubulin served as the control for loading. Experiments were repeated in triplicate. Data represent mean ± S.E; n = 3. ** Represents *p* < 0.01 compared to control group.

To investigate how autophagy affects GC survival during oxidative injury, we treated cells with the autophagy inhibitor, 3-MA. As shown in Figure 2a, the loss of GC viability upon H_2_O_2_ exposure was markedly reversed in GCs pretreated with 3-MA (Figure 2a). Consistent with this, MEL exhibited similar level of inhibition on cell death caused by oxidative stress (Figure 2b). Our previous studies showed that exposing to H_2_O_2_ with a relative long-time initiated GC apoptosis. We thus examined whether there was any correlation between MEL-mediated autophagy and apoptosis in the early stages of cell death induced by H_2_O_2_. Both MEL and 3-MA prevented oxidative stress-induced loss of GC viability. However, the treatment with Z-VAD-FMK, a caspase inhibitor, showed no effect on the cell viability of H_2_O_2_-treated GCs (Figure 2c). Similarly, the results of Western blotting showed that the level of caspase-3 did not significantly change in GCs treated with H_2_O_2_ and/or MEL (Figure 2d,e).

### 3.3. MEL Inhibits Mitochondria Injury in H_2_O_2_-Treated GCs

**Figure 3:**
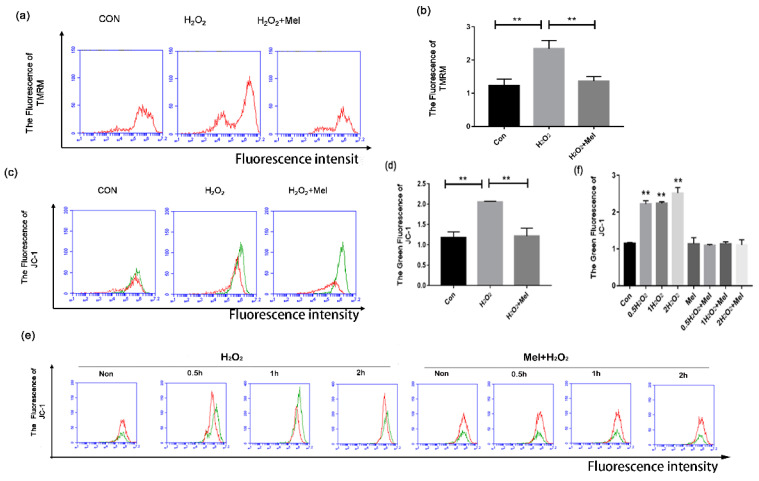
MEL alleviates oxidative damage in GC by inhibiting mitochondria. (**a**,**b**) GCs harvested from ovarian follicles were exposed to 200 μ M H_2_O_2_ for 1 h, and then treated with 10 μM MEL for 2 h. Mitochondria were stained with TMRM (red) and analyzed by FCM. (**c**,**d**) Mitochondrial membrane potential was monitored by a mitochondrial-specific dual fluorescence probe. GCs were treated with 200 μ M H_2_O_2_ for 1 h and incubated with 10 μM MEL for 2 h. Then, cells were incubated with JC-1 for 20 min and detected by FCM. (**e**,**f**) GCs treated with H_2_O_2_ for 1 h were then cultured with 10 μM MEL, and 0, 0.5,1 or 2 h later, JC-1 fluorescence was detected using FCM analysis. The +Y axis is 100 times the number of cells, and the +X axis is the intensity of cell fluorescence. Experiments were repeated in triplicate. Data represent mean ± S.E; *n* = 3. ** Represents *p* < 0.01 compared to control group.

Mitophagy has been reported to instigate programmed cell death [25]. Using tetramethyl rhodamine ethyl ester (TMRM) staining assay, we observed a significant increase in the fraction of depolarized mitochondria during H_2_O_2_ incubation, which was significantly reduced through MEL supplementation (Figure 3a,b). To further evaluated the mitochondrial damage, we next stained GCs with JC-1, a specific fluorescent probe for detecting mitochondrial membrane potential (MMP, Δψm). Since mitochondrial damage induced MMP reduction, JC-1 cannot concentrate in the matrix, resulting in turning a J-aggregate into a monomer and producing green fluorescence which could be tested by flow cytometry. The result showed a significant reduction of functional mitochondria in GCs with H_2_O_2_ treatment, while MEL produced a significant mitigating effect on mitochondrial depolarization during oxidative stress (Figure 3c,d). The protective effects of MEL on mitochondria injury were detected in GCs with H_2_O_2_ exposure for 0.5–2 h (Figure 3e,f).

### 3.4. MEL Repressed Oxidative Stress-Induced Mitophagy in GCs

**Figure 4:**
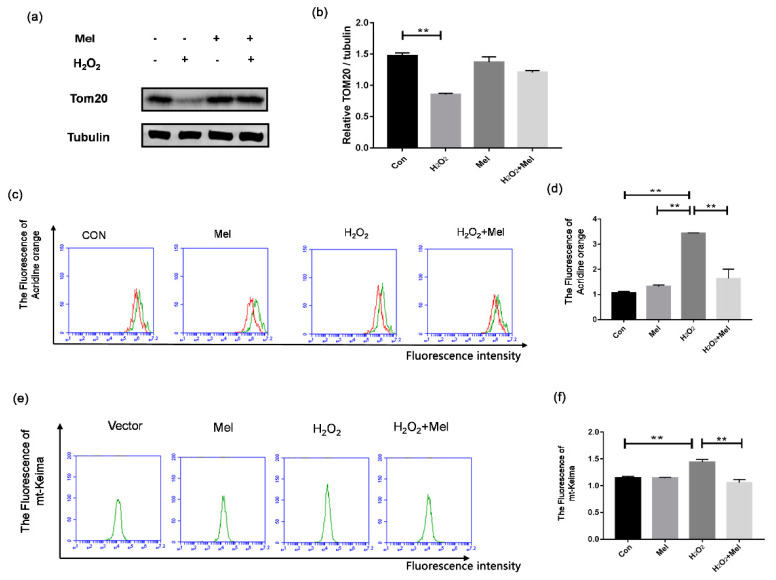
MEL suppressed oxidative stress-induced mitophagy in GCs. (**a**,**b**) GCs incubated with or without 200 μM H_2_O_2_ for 1 h were then treated with MEL for another 2 h. The protein expression of Tom20 was determined by Western blotting and α-tubulin served as the control for loading. (**c**,**d**) GCs were exposed to 200 μM H_2_O_2_ for 1 h, and then treated with 10 μM MEL for 2 h. The acridine orange staining was performed to detect acidic autophagic vacuoles. (**e**,**f**) GCs were transfected with pMT-mKeima-Red after exposure to 200 μM H_2_O_2_ for 1 h, and then treated with 10 μM MEL for 2 h. Then, the cells were washed twice with PBS and detected by FCM analysis. The +Y axis is 100 times the number of cells, and the +X axis is the intensity of cell fluorescence. Experiments were repeated in triplicate. Data represent mean ± S.E; *n* = 3. Significances were marked as ** *p* < 0.01 vs. control group.

To examine whether MEL alleviates mitochondrial damage by inhibiting mitophagy, we also tested the indicators related to mitophagy. Through Western blot assay, we demonstrated that H_2_O_2_ treatment reduced the level of TOM20, which is a mitochondrial marker protein for evaluating the mitochondrial mass. By contrast, addition of MEL prevented the downregulation of TOM20 level in H_2_O_2_-treated cells (Figure 4a,b). Using acridine orange staining, we found that MEL treatment significantly attenuated the formation of autolysosomes induced by H_2_O_2_ stimulating (Figure 4c,d). To visually reflect the degree of mitophagy, we constructed an expression vector of keima located in mitochondria (MT-keima) and transfected it into GCs. The result of flow cytometry showed that MEL reduced fluorescence intensity of MT-keima in the H_2_O_2_-treated group (Figure 4e,f).

### 3.5. MEL-Induced Mitigation of Mitophagy Reduces Cellular Oxidative Damage

**Figure 5:**
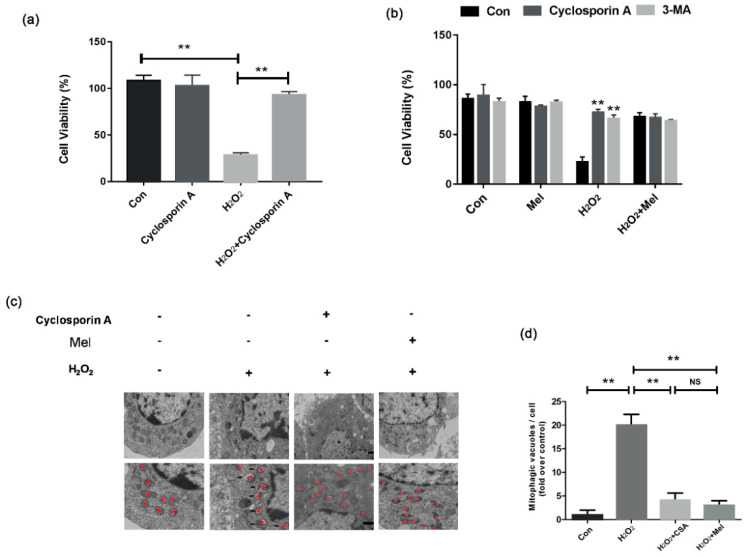
MEL improved GCs survival by inhibiting mitochondrial autophagy. (**a**) GCs incubated with 200 μM H_2_O_2_ for 1 h and treated with 10 μ M Cyclosporin A for 2 h. Cell viability was detected using the CCK-8 assay. (**b**) GCs incubated with 200 μM H_2_O_2_ for 1 h were treated with 10 μM Cyclosporin A or 50 μM 3-MA with or without 10 μM MEL. Two hours later, cell viability was detected using the CCK-8 assay. (**c,d**) GCs pretreated with 200 μM H_2_O_2_ for 1 h were then treated with 10 μM Cyclosporin A or 10 μM MEL. One hour later, cells were collected for TEM imaging of the mitophagic vacuoles and counted the number of mitophagic vacuoles per cell section in GCs. Experiments were repeated in triplicate. Data represent mean ± S.E; *n* = 3. Significances were marked as ** *p* < 0.01 vs. control group.

To evaluate the effect of MEL-regulated mitophagy on cell viability during oxidative stress, GCs were treated with the mitophagy inhibitor cyclosporine A (CsA) and autophagy inhibitor 3-Methyladenine (3-MA). As shown in Figure 5a, the reduction in cell viability caused by H_2_O_2_ was significantly alleviated by CsA treatment (Figure 5a). In accordance with this, cells treated with MEL and 3-MA showed similar level of inhibitory effect on the GCs’ death during H_2_O_2_ incubation (Figure 5b). Using electron microscopy, we observed that both MEL and CsA reduced the number of mitochondria-containing autophagic vacuoles in H_2_O_2_-treated GCs.

### 3.6. MEL Prevents GCs’ Oxidative Injury by Inhibiting Mitophagy though the PINK-Parkin Pathway

**Figure 6:**
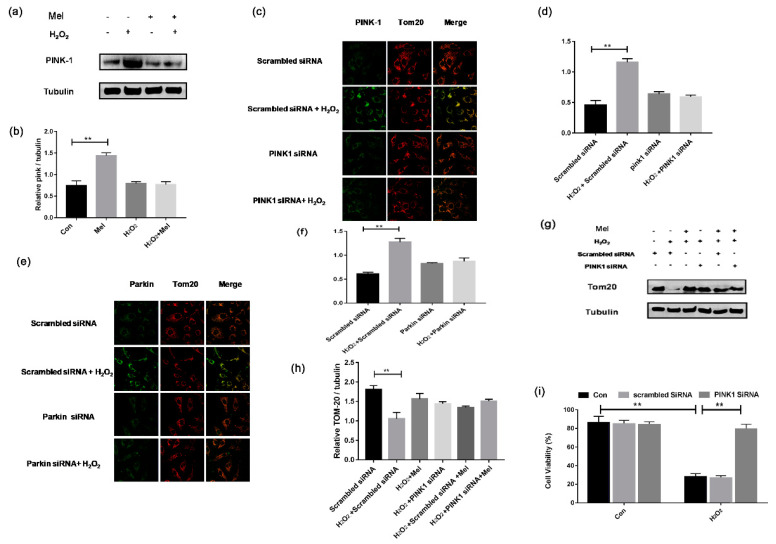
MEL relieved mitophagy and protected GCs from oxidative injury by targeting PINK-Parkin pathway. (**a**,**b**) Western blot assay of PINK1. α-tubulin was used as an invariant control for equal loading. (**c**–**f**) GCs were transfected with PINK siRNA or Parkin siRNA for 24 h. Scramble control siRNA was transfected cells were served as control group. After incubating with 200 μM H_2_O_2_ for 1 h, cells were cultured in media containing 10 μM MEL for another 2 h. Mitochondria were stained by anti-Tom20 (red) and the Parkin or PINK1 was counterstained with anti-Parkin or anti-PINK1 (green). (**g**,**h**) GCs transfected with PINK1 siRNA or scramble control siRNA for 24 h were exposed to 200 μM H_2_O_2_ for 1 h and treated with 10 μM MT for another 2 h. α-tubulin was used as an invariant control for equal loading. (**i**) GCs transfected with PINK1 siRNA or scramble control siRNA for 24 h were exposed to 200 μ M H_2_O_2_ for 1 h. Cell viability was determined using the CCK-8 assay. Experiments were repeated in triplicate. Data represent mean ± S.E; *n* = 3. Significances were marked as ** *p* < 0.01 vs. control group.

PINK1-Parkin is one of the mitophagy pathways that has been described previously [22]. To further clarify the regulatory mechanism of mitophagy in MEL-treated GCs, we detected the expression of PINK1 and Parkin. Western blotting assay showed a significant increase in PINK1 expression in GCs subjected to H_2_O_2_ exposure, which was reduced following MEL treatment (Figure 6a,b). To confirm this result, GCs were transfected with siRNAs against PINK1 and Parkin. The results showed that blocking PINK1-Parkin significantly reduced the loss of mitochondrial mass during oxidative stress, as indicated by TOM20 level (Figure 6c–f). Using Western blotting assay, we further demonstrated that both PINK1 knockdown and MEL treatment had the same effect on preventing H_2_O_2_-induced mitochondrial loss (Figure 6g,h). We next performed CCK-8 assay to detect cell viability. Figure 6i shows that PINK1 knockdown significantly alleviates the decrease in GC viability upon oxidative exposure.

## 4. Discussion

Ovarian aging is often accompanied by an elevation of ROS level and dysfunction of mitochondria in follicular GCs [22]. Melatonin (MEL) is a well-recognized antioxidant and free radical scavenger that enriched in the follicular fluid [26]. Evidence has emerged that MEL could protect the cellular components of ovarian GCs from oxidative injury [27], although the underlying mechanism has not been fully elucidated. In this study, we demonstrated the role of MEL in antagonizing oxidative GC injury by inhibiting mitophagy.

Autophagy is a catabolic process that degrades metabolic waste to avoid cellular damage caused by adverse conditions such as nutrient deficiency and oxidative stress [28]. Therefore, autophagy is necessary for the normal cell function in the basic state. However, an excess of autophagy might eventually induce cell death due to the excessive digestion of intracellular components. According to the types of degraded organelles, autophagy can be divided into selective autophagy and non-selective autophagy. Mitochondrial autophagy is a specific autophagy phenomenon, which selectively clears damaged or redundant mitochondria through autophagy lysosome. The current study found that the inhibition of mitophagy by MEL could mitigate oxidative stress-induced GC death. Therefore, our data suggested that MEL might inhibit a lethal form of mitophagy in GCs suffering oxidative damage.

The endogenous ROS is produced though two pathways: one is from various metabolic process; the other is from the electronic transport system. Mitochondria are closely related to cellular respiration and metabolism, so mitochondria are the main generator of endogenous ROS as well as the main target organelle for oxidative damage. Mitochondrial permeability transfer pore (MPTP) is a kind of non-selective compound pore composed of various proteins located between the inner and outer membrane of mitochondria. Its periodic opening plays an important role in maintaining the electrochemical equilibrium in mitochondria. It has been proven that many factors can affect the opening of MPTP pores. ROS can activate the opening of non-specific MPTP on mitochondria. The opening of MPTP pores allows the substrate of respiratory chain to reach equilibrium between cytoplasm and matrix, which makes the H+ gradient inside and outside mitochondria disappear, leading to the decrease in mitochondrial membrane potential (ΔΨm) and depolarization. Therefore, mitochondrial membrane depolarization can reflect the open state of MPTP pores. Once the MPTP pore is opened, the permeability of mitochondrial membrane is increased, which will lead to the activation of apoptosis, mitophagy and autophagosome formation. Mitochondrial complex I inhibition triggers a mitophagy-dependent ROS increase, leading to necroptosis and ferroptosis in melanoma cells [29,30,31]. As reported, the treatment of melatonin can inhibit the depolarization of mitochondrial membrane during H_2_O_2_ treatment [32], which is in agreement with our data. This study also found that melatonin-mediated GCs protection is dependent on the suppression of mitophagic death rather than apoptosis. Therefore, our results provided the possibility that melatonin could inhibit oxidative stress-induced mitophagy of granulosa cells by ameliorating mitochondrial damage.

Mitophagy could be divided into two types. One is PINK1-Parkin-dependent mitophagy, the other is PINK1-Parkin independent mitophagy. The latter was mediated by FUDC1 (Fun14 domain containing 1) and BNIP3L/NIX (BCL2 intervening protein 3-like/NIP3-like protein X). Recent studies have shown that the synergism of PINK1, Parkin and ubiquitin to regulate mitophagy plays an important role in the quality control and the maintenance of normal function of mitochondria. PTEN-induced putative kinase 1 (PINK1) is a protein kinase located on the mitochondrial membrane. Under external stimulus, PINK1 was positioned on OMM, which promoted Parkin’s recruitment [20]. Parkin ubiquitinates several outer membrane components, and the polyubiquitin chain is subsequently phosphorylated by PINK1 as a signal for the autophagy mechanism [33]. Adapter proteins (p62, OPTN, NDP52) recognize phosphorylated polyubiquitin chains on mitochondrial proteins and initiate autophagosome formation by binding to LC3 [34,35]. In this study, we demonstrated that MEL (1) inhibited mitophagy through the PINK1-Parkin pathway, and (2) reduced oxidative damage of GCs by repressing PINK1-Parkin-mediated mitophagy. Collectively, this work may bring forward new mechanism involving PINK1-Parkin-mitophagy axis for MEL-mediated GC protection.

N-acetyl-5-methoxytryptomine (melatonin) is a hormone secreted by the pineal gland that serves as a key regulator of circadian rhythms. In addition to the pineal gland, melatonin is secreted by many organs, such as the brain, gastrointestinal tract, retina, uterus and ovaries. High concentrations of melatonin were also detected in follicular fluid (FF). Further researches revealed that most of melatonin in FF is derived from the blood. Melatonin secretion is controlled by the endogenous circadian clock in the suprachiasmatic nucleus in response to length of daylight [36]. The disturbance of circadian rhythm could in turn lead to abnormal secretion of melatonin [37]. In fact, studies have shown that patients with follicular developmental disorders such as polycystic ovary syndrome are often associated with circadian rhythm disorders and abnormal melatonin secretion. On the other hand, melatonin has been shown to inhibit the oxidative damage in GCs, which is essential for the normal development and function of follicles [38]. Studies have demonstrated that most of the melatonin presented in ovarian follicles is derived from the circulatory system [39,40]. Since melatonin serves as a hormone with endocrine actions, our current study suggested that the disturbed secretion of melatonin caused by certain sleep disorders, such as insomnia, might impede the normal development of ovarian follicles. In conclusion, this study indicated that MEL could suppress mitophagy though PINK1-Parkin pathway to rescue GCs from H_2_O_2_-induced oxidative injury. By this way, MEL treatment might provide benefits to clinical therapy for anovulation-related female infertility.

## Data Availability

Data available in a publicly accessible repository.
